# Thermodynamic Modeling and Validation of the Temperature Influence in Ternary Phase Polymer Systems

**DOI:** 10.3390/polym13050678

**Published:** 2021-02-24

**Authors:** Marta Romay, Nazely Diban, Ane Urtiaga

**Affiliations:** Department of Chemical and Biomolecular Engineering, ETSIIyT, University of Cantabria, Avda. Los Castros s/n, 39005 Santander, Spain; romaym@unican.es (M.R.); urtiaga@unican.es (A.U.)

**Keywords:** Flory–Huggins theory, binodal curve, temperature influence, ternary interaction, Hansen solubility parameter, polyvinylidene fluoride (PVDF), polyethersulfone (PES)

## Abstract

The effect of the temperature, as a process variable in the fabrication of polymeric membranes by the non-solvent induced phase separation (NIPS) technique, has been scarcely studied. In the present work, we studied the influence of temperature, working at 293, 313 and 333 K, on the experimental binodal curves of four ternary systems composed of PVDF and PES as the polymers, DMAc and NMP as the solvents and water as the non-solvent. The increase of the temperature caused an increase on the solubility gap of the ternary system, as expected. The shift of the binodal curve with the temperature was more evident in PVDF systems than in PES systems indicating the influence of the rubbery or glassy state of the polymer on the thermodynamics of phase separation. As a novelty, the present work has introduced the temperature influence on the Flory–Huggins model to fit the experimental cloud points. Binary interaction parameters were calculated as a function of the temperature: (i) non-solvent/solvent (*g_12_*) expressions with UNIFAC-Dortmund methodology and (ii) non-solvent/polymer (*χ_13_*) and solvent/polymer (*χ_23_*) using Hansen solubility parameters. Additionally, the effect of the ternary interaction term was not negligible in the model. Estimated ternary interaction parameters (*χ_123_*) presented a linear relation with temperature and negative values, indicating that the solubility of the polymers in mixtures of solvent/non-solvent was higher than expected for single binary interaction. Finally, PES ternary systems exhibited higher influence of the ternary interaction parameter than PVDF systems.

## 1. Introduction

Non-solvent induced phase inversion (NIPS) is a synthesis technique commonly used to manufacture asymmetric polymeric membranes for separation processes [1], both in laboratory and industrial scale [2,3]. The mechanism of membrane formation using NIPS consists in the precipitation of a polymer solution when it is introduced in a non-solvent bath by the exchange between solvent and non-solvent or coagulant [4]. The liquid–liquid demixing that occurs in the NIPS process, and ultimately the membrane structure, are strongly affected by the thermodynamics and kinetics of the system.

**Figure 1:**
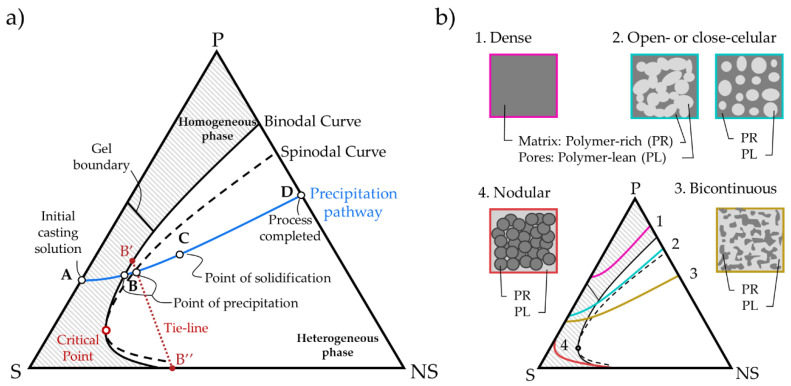
(**a**) Theoretical ternary phase diagram representing the polymer (P), the solvent (S) and the non-solvent (NS) compositions and (**b**) structures obtained depending on the precipitation pathway. Adapted from [5,6].

Ternary phase diagrams, which are temperature dependent, are employed to depict the thermodynamics and kinetic characteristics of a certain NIPS process. Figure 1 shows a ternary diagram in which the continuous and dashed blue curves represent the thermodynamic binodal and spinodal curves, respectively. The area between both curves is called the metastable region. The point where these curves meet is the critical point. The binodal curve defines the boundary between a homogeneous phase and a heterogeneous phase. When the precipitation pathway (yellow line) crosses the binodal curve (point B) the polymer nucleation takes place and the polymer solution separates into two equilibrium phases, polymer-rich (B′) and polymer-lean (B″) phases. These equilibrium compositions are located on the binodal curve and depict a tie-line in the ternary phase diagram. The pathway between points B-C defines the precipitation of the entire membrane, and finally, from C to D points the rest of the solvent is exchanged with the non-solvent.

The final membrane morphology is described by the distribution and shape of the pores created in the NIPS process. The position of the binodal curve and the rate of solvent/non-solvent exchange modify the pore shape. When the binodal curve is located near the polymer–solvent axis, the precipitation pathway reaches more rapidly the binodal curve so big finger-like pores are expected. Otherwise, when the binodal curve is displaced to the right side of the diagram, smaller sponge-like pores are obtained. The entry point (B) of the precipitation pathway into the heterogeneous phase affects the pore distribution. Depending on the pathway followed during solvent/non-solvent exchange different solid phase morphologies can occur (Figure 1b), i.e., dense membranes (pathway 1) were obtained when the pathway crosses the gel homogeneous region, at sufficiently high initial polymer concentration causing slow exchange due to high viscosity; binodal decomposition occurs if the precipitation pathway enters the heterogeneous phase through the metastable region, to form (pathway 2) open-cellular or closed-cellular membrane morphologies, or (pathway 4) nodular pore distributions; otherwise, spinodal decomposition takes place if the precipitation pathway enters the heterogeneous phase through the heterogeneous phase, obtaining (pathway 3) bicontinuous (lacy) distribution of pores [6]. It is deemed that, due to the significant dependence of membrane morphology on thermodynamics and kinetics of the ternary phase systems, the development of mathematical models able to predict the thermodynamic and kinetic characteristics of different polymer/solvent/non-solvent systems would facilitate the decision on the selection of membrane synthesis variables, such as initial polymer concentration, process temperature or even solvent/non-solvent combination.

Different mathematical models have been developed to describe the thermodynamics of ternary systems, such as Flory–Huggins (FH) and compressible regular solution theory (CRS) [7,8]. FH theory, characterized by its relative mathematical simplicity and good prediction of phase behavior [9], is based on the Gibbs free energy of a mixture and the use of binary interaction parameters. One of the limitations of the FH model is that the binary interaction parameters are traditionally obtained in experimental assays. Many efforts have been made to obtain these parameters from tabulated solubility parameters. Hansen’s solubility parameters are commonly used because of the huge amount of data available for polymers and solvents. However, solubility parameters for new materials (i.e., green solvents or copolymers) are seldom accessible and need to be calculated with group contribution methods such as Hoftyzer and Van Krevelen [10], Hoy [10], Just [11] or Stefanis [12]. Nevertheless, solubility parameters calculated with this methodology do not give accurate predictions yet.

Polyethersulfone (PES) and polyvinylidene fluoride (PVDF) are polymers classically used to manufacture membranes for different industrial separation applications. Particularly PVDF is a polymer with interest to be used for the development of photocatalytic membranes [4]. Temperature plays an important role in the manufacture of polymeric membranes. From the thermodynamic viewpoint, increasing the temperature of the ternary system will increase the demixing gap [2]. Meanwhile, temperature varies the diffusion parameters of the solvent and non-solvent and therefore, the kinetics of exchange between them. Thus changes in the processing temperature affect the final membrane morphology [1]. Despite the fact that during the industrial synthesis of polymeric membranes the temperature of the polymer solution or the coagulation bath is often set different than room temperature, few works have addressed the experimental analysis of the effect of temperature on the thermodynamics of ternary systems [6,13]. Furthermore, there is a lack of validations of predictive models considering temperature for PVDF and few works address the temperature effect for the synthesis of PES membranes. Kahrs et al., evaluated the influence of temperature on the thermodynamics of PES ternary systems [6,13]. When they compared PES membranes fabricated with N-methyl pyrrolidone (NMP) and 2-pyrrolidone (2Pyr) as the solvents, the SEM images showed an increase in the number of pores when the temperature was increased from 20 to 40 °C, because the reduction of the viscosity favored the nuclei formation in the lean-polymer phase [6].

The aim of this work is on the one hand, to develop an enhanced thermodynamic model based on Flory–Huggins theory that incorporates the effect of temperature to predict the binodal curves of different ternary phase diagrams. On the other hand, the model was validated experimentally, considering four ternary systems, using PVDF and PES as the polymers; *N*,*N*-dimethylacetamide (DMAc) and *N*-methyl pyrrolidone (NMP) as the solvents; and water as the non-solvent/coagulant, at three temperatures in the range 293–313 K. The importance of the binary and ternary interaction parameters on the model predictions was explored and discussed. Additionally, a potential influence of the solid polymer state (rubbery or glassy) on behavior of the thermodynamics of the ternary systems at different temperatures was observed.

## 2. Theoretical Section

### 2.1. Thermodynamic Model

In this study, Flory–Huggins theory was selected to describe the thermodynamic model of a ternary system. FH theory is based on the Gibbs free energy of a mixture (Δ*G_M_*), Equation (1). The last term, that represents the ternary interactions of the components, is usually neglected to simplify the calculations [14]. In this work, the effect of neglecting this term will also be addressed.

(1) $$\frac{\Delta G_{M}}{RT}{\;=\;n}_{1}\;\ln\phi_{1}{+n}_{2}\;\ln\phi_{2}{+n}_{3}\;\ln\phi_{3}{+g}_{12}n_{1}\phi_{2}{+\;\chi }_{13}n_{1}\phi_{3}{+\chi }_{23}n_{2}\phi_{3}{+\chi }_{123}n_{1}\phi_{2}\phi_{3}$$

In Equation (1), the subscripts 1, 2 and 3 refer to the non-solvent, solvent and polymer, respectively. Equation (2) defines the molar volume fraction (*ϕ_i_*) as function of the number of moles (*n_i_*) and the molar volume (*V_i_*) of the component *i*.

(2) $$\phi_{i}\;=\;\frac{n_{i}V_{i}}{n_{1}V_{1}{+n}_{2}V_{2}{+n}_{3}V_{3}}$$

The binary interaction parameters (*g_ij_* or *χ_ij_*) represent the interaction between each pair of components. Solvent–polymer (*χ_23_*) and non-solvent–polymer (*χ_13_*) are interaction parameters independent of the concentration. The non-solvent–solvent interaction parameter (*g_12_*) is a solvent concentration-dependent parameter, function of *u_2_*, which is defined in Equation (7). The ternary interaction parameter (*χ_123_*) was estimated through fitting modeled curves to the experimental cloud points [15].

The equilibrium phases, *B*′ and *B*″, connected by the tie-line have the same chemical potential for each component (*μ_i_*), Equation (3), but differ in composition. The chemical potential of each component, Equations (4)–(6), is derived from Equation (1).

(3) $$\Delta \mu_{i}^{\prime }\;=\;\Delta \mu_{i}^{\prime \prime }$$

(4) $$\frac{\Delta \mu_{1}}{RT}\;=\;\ln\phi_{1}+1-\phi_{1}-s\phi_{2}-r\phi_{3}+\left(g_{12}\phi_{2}{+\chi }_{13}\phi_{3}\right)\left(\phi_{2}+\phi_{3}\right)-\chi_{23}s\phi_{2}\phi_{3}-u_{1}u_{2}\phi_{2}\left(\frac{{dg}_{12}}{{du}_{2}}\right){+\chi }_{123}\phi_{2}\phi_{3}\left(1-2\phi_{1}\right)$$

(5) $$s\frac{\Delta \mu_{2}}{RT}\;=\;\mathrm{sln}\phi_{2}+s-s\phi_{2}-\phi_{1}-r\phi_{3}+\left(g_{12}\phi_{1}{+\chi }_{23}s\phi_{3}\right)\left(\phi_{1}+\phi_{3}\right)-\chi_{13}\phi_{1}\phi_{3}{+u}_{1}u_{2}\phi_{1}\left(\frac{{dg}_{12}}{{du}_{2}}\right){+\chi }_{123}\phi_{1}\phi_{3}\left(1-2\phi_{2}\right)$$

(6) $$r\frac{\Delta \mu_{3}}{RT}\;=\;\mathrm{rln}\phi_{3}+r-r\phi_{3}-\phi_{1\;}-s\phi_{2}+\left(\chi_{13}\phi_{1}{+s\chi }_{23}\phi_{2}\right)\left(\phi_{1}+\phi_{2}\right)-g_{12}\phi_{1}\phi_{2}{+\;\chi }_{123}\phi_{1}\phi_{2}\left(1-2\phi_{3}\right)\;$$

(7) $$u_{i}\;=\;\frac{\phi_{i}}{\phi_{1}+\phi_{2}}$$

In the above equations, *s* and *r* are the molar volume relations *V_1_/V_2_* and *V_1_/V_3_*, respectively. It has been reported that at sufficiently high molecular weight of the polymer the phase diagram is fairly insensitive to the choice of *V_3_* (polymer molar volume) provided that *V_1_/V_3_* is sufficiently small [16]. The last term of Equations (4)–(6) corresponds to the partial derivative of the term for ternary interaction. Most of the literature neglects ternary interaction among the components. In this work, the ternary interaction term will be evaluated under two hypotheses: (1) omitting it or (2) considering it.

### 2.2. Influence of the Temperature in the Model

**Figure 2:**
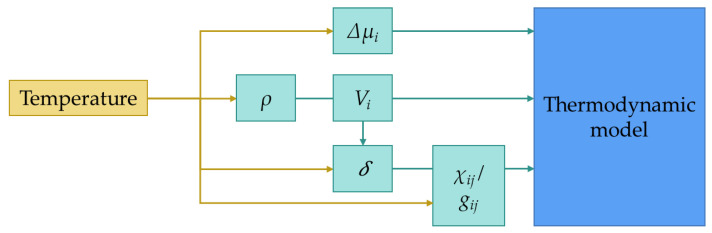
Influence of the temperature on different parameters that are part of the thermodynamic model.

Temperature is an important variable of the phase inversion process that affects the chemical potential, the solubility parameters (*δ*), the binary interaction parameters, and the components density (*ρ*) and thus their molar volume. The next diagram (Figure 2) schematizes the effect of temperature over the mentioned functions and parameters.

The temperature is directly introduced in the expression of chemical potentials. The molar volume (*V_i_*) changes with the temperature because of the temperature dependence of the density (*ρ*), Equation (8). Density values of the solvents and non-solvent at different temperatures were retrieved from the database of the software Aspen Plus V9. Table 1 compiles the relevant data and properties of the solvents and non-solvent for the model in this study.

(8) $$V_{i}(T)\;=\;\frac{\rho (T)}{M_{w}}$$

Over the years, the authors have deepened in the concentration dependence of the binary interaction parameters, which initially were considered constant. Later, Altena and Smolders [17] determined the concentration dependency of *g_12_*, and Yilmaz et al. [18] analyzed the influence of concentration on *χ*_23_, although both studies agreed to consider *χ*_13_ as constant parameter. Currently, it is widely agreed that only *g_12_* is a concentration-dependent parameter [15,19,20,21,22].

Interaction parameters *χ_13_* and *χ_23_* can be obtained experimentally; light scattering, vapor pressure depression and membrane osmometry are techniques commonly proposed to determine *χ_23_*, and equilibrium swelling for *χ_13_*. However, reliable estimations can be obtained using Equation (9), defined by Hansen [23] and widely used in the literature [15,24,25,26,27,28,29]. This expression uses Hansen solubility parameters that are composed of three contributions: dispersive (*δ_d_*), polar (*δ_p_*) and hydrogen bonding (*δ_h_*). Equation (9) also includes the correction factor (*α_ij_*).

(9) $$\chi_{ij}{\;=\;\alpha }_{ij}\frac{V_{i}}{RT}\left[{\left(\delta_{i,d}\;-{\;\delta }_{j,d}\right)}^{2}+0.25{\left(\delta_{i,p}\;-{\;\delta }_{j,p}\right)}^{2}+0.25{\left(\delta_{i,h}\;{\;\delta }_{j,h}\right)}^{2}\right]$$

Table 2 compiles the Hansen’s solubility parameters for the polymers and solvents of interest in the present study, at the reference temperature of 298 K [23]. 

The dependency of solubility parameters with the temperature can be calculated with Equations (10)–(12) [23]. Molar volumes (*V* and *V_ref_*, at the reference temperature of 298 K) for solvents and non-solvent can be found in Table 1. For polymers, the molar volumes in Equations (10)–(12) refer to the molar volume of the monomer (V* and V*_ref_) calculated using Equations (13) and (14) [10]. Equation (13) is employed for the rubbery polymer and Equation (14) for glassy polymers. These equations are function of the van der Waals volume (*V_w_*), the temperature and, in the case of glassy polymers, the glass transition temperature (*T_g_*).

(10) $$\delta_{d}{\;=\;\delta }_{d,ref}{\left(\frac{V_{ref}}{V}\right)}^{1.25}$$

(11) $$\delta_{p}{\;=\;\delta }_{p,ref}{\left(\frac{V_{ref}}{V}\right)}^{0.5}$$

(12) $$\delta_{h}{\;=\;\delta }_{h,ref}/\exp\left[-{1.22\cdot 10}^{-3}\left(T_{ref}\;-T\right)-\ln\left({\left(\frac{V_{ref}}{V}\right)}^{0.5}\right)\right]$$

(13) $$\;Rubbery\;\to {\;V}_{i}^{*}\left(T\right){\;=\;V}_{w}\left[{1.30+10}^{-3}T\right]$$

(14) $$Glassy\;\to {\;V}_{i}^{*}\;\left(T\right){\;=\;V}_{w}\left[1.30+{0.55\;10}^{-3}T_{g}\;+\;{0.45\;10}^{-3}T\right]$$

Table 3 compiles the chemical structure and parameters relevant for the mathematical model of the monomer unit of the polymers.

Equation (18) presents the effect of concentration on the binary interaction parameter between solvent and non-solvent. It is obtained from the relationship, Equation (15), between the excess of Gibbs free energy (*G^E^*), Equation (16), and the Gibbs free energy for a binary mixture (Δ*G_M_*), Equation (17) [30].

(15) $$\Delta G_{M}\;=\;\Delta G_{M,\;ideal}{+G}^{E}\;=\;RT\sum_{i=1}^{\;N}x_{i}{\ln x}_{i}{+G}^{E}$$

(16) $$\frac{G^{E}}{RT}{\;=\;x}_{1}{\ln\gamma }_{1}{+x}_{2}{\ln\gamma }_{2}$$

(17) $$\frac{\Delta G_{M}}{RT}{\;=\;x}_{1}\ln\phi_{1}{+x}_{2}\ln\phi_{2}{+g}_{12}x_{1}\phi_{2}$$

(18) $$g_{12}\;=\;\left(\frac{G^{E}}{RT}{+x}_{1}{\ln x}_{1}{+x}_{2}{\ln x}_{2}{-x}_{1}\ln\phi_{1}{-x}_{2}\ln\phi_{2}\right)/x_{1}\phi_{2}$$

*G^E^* is calculated from the activity coefficient (*γ*) of vapor–liquid equilibrium data, obtained with the modified UNIFAC-Dortmund methodology based on groups contribution of the activity coefficients [31]. This methodology is described in Appendix A. A polynomial expression suggested by Tompa [32] was used to fit the *g_12_* as a function of the solvent concentration, Equation (19).

(19) $${\;g}_{12}\left(\phi_{2}\right){\;=\;a}_{o}{+a}_{1}\phi_{2}{+a}_{2}\phi_{2}^{2}{+a}_{3}\phi_{2}^{3}\ldots$$

### 2.3. Calculation Procedure

In this model there are six variables corresponding to the volume fraction of the three components in the two phases. The set of equations is formed by the three equilibrium relationships for each component (Equation (3)) and the two mass balance equations in each phase, Equation (20).

(20) $$\sum_{i=1}^{3}\phi_{i}^{\prime }\;=\;\sum_{i=1}^{3}\phi_{i}^{\prime \prime }\;=\;1$$

The system of equations is solved using KNITRO solver in GAMS Development Corporation 27.3.0, USA by minimizing the objective function *F*, Equation (21), as the equilibrium is found when the chemical potential of each component is the same in both phases. The polymer lean-phase molar volume fraction (*ϕ_3_^′^*) is selected as an independent variable.

(21) $$F\;=\;{\left({\Delta \mu }_{1}^{\prime }{-\Delta \mu }_{1}^{\prime \prime }\right)}^{2}+{\left[s\;\left({\Delta \mu }_{2}^{\prime }{-\Delta \mu }_{2}^{\prime \prime }\right)\right]}^{2}+{\left[r\;\left({\Delta \mu }_{3}^{\prime }{-\Delta \mu }_{3}^{\prime \prime }\right)\right]}^{2}$$

As a summary, Table 4 collects the physical parameters of the mathematical model that have been calculated or estimated by fitting to the experimental cloud points. 

## 3. Materials and Methods

### 3.1. Materials

The polymers used were polyvinylidene fluoride (PVDF) Kynar^®^ 761A (Arkema Inc., Colombes, France) with melt viscosity of 32 Kps@100s^−1^ and polyethersulfone (PES) Sumikaexcel^®^ 5200P (Sumitomo Chemical Europe Inc., Machelen, Belgium) with a reduced viscosity of 0.52 (1 (*w*/*v*)% PES dissolved in DMF). The viscosity data of the polymers are supplied by the companies. *N*,*N*-Dimethylacetamide (DMAc) and *N*-methyl-2-pyrrolidone (NMP) (Acros Organics) were used as solvents and ultrapure water was used as the non-solvent.

### 3.2. Cloud Point

The binodal curve is experimentally obtained by cloud point titration. Cloud point experiments were performed for both solvents (DMAc and NMP) using water as the non-solvent and PVDF and PES as the polymers. First, polymers were dried in an oven overnight at 333 K. Then, polymer–solvent binary mixtures were prepared at several polymer concentrations. Polymeric solutions, of an initial volume of 60 mL, were kept under constant magnetic stirring. Drops of water were added using a digital burette (Tittrete^®^, Brand GMBH + CO KG, Wertheim, Germany) to a polymer solution until permanent turbidity was obtained for 1 h. Experiments were performed in duplicate at different polymer concentrations in the range between 2 and 15 wt% using DMAc and NMP as solvents. Higher polymer concentrations were not tested as the high viscosity of the solution hindered its adequate stirring. The influence of the temperature was studied at 293, 313 and 333 K. Student’s *t*-test was used to statistically compare the significance of the difference between the experimental cloud points of each ternary system at the three temperatures.

## 4. Results and Discussion

### 4.1. Experimental Cloud Point

**Figure 3:**
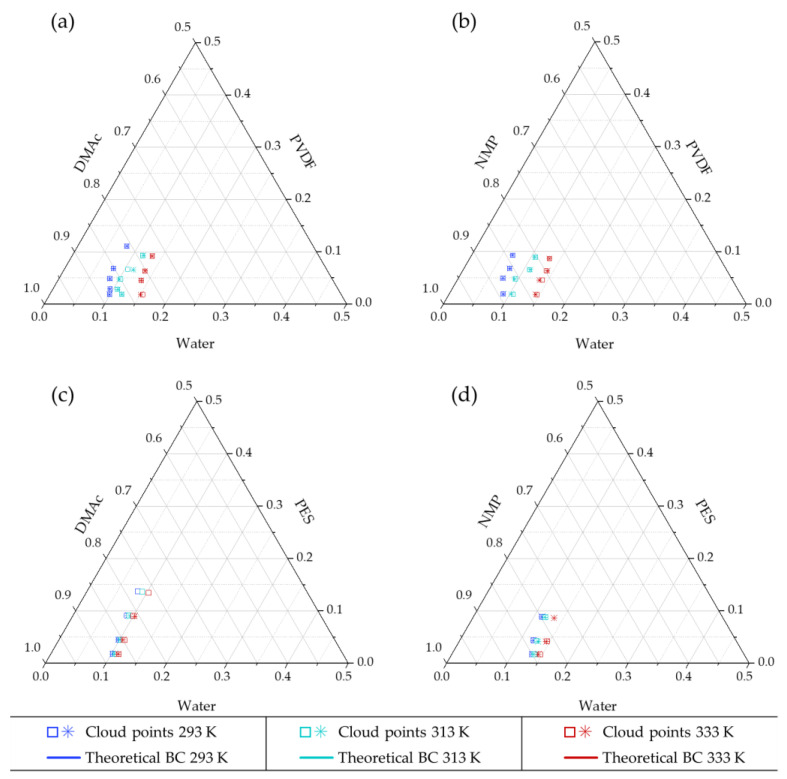
Cloud points at 293, 313 and 333 K of (**a**) PVDF–DMAc–water, (**b**) PVDF–NMP–water, (**c**) PES–DMAc–water and (**d**) PES–NMP–water systems. Duplicated experimental results are represented by □ and *. Significant statistical difference with Student’s *t*-test (*p* < 0.05) was found in all systems comparing points between 293 and 313 K and between 313 and 333 K. Significant difference was also found at 293 K between systems comparing the use of DMAc and NMP with the same polymer and between PVDF and PES with the same solvent.

Cloud point results are represented in Figure 3 and Table A3 of Appendix A compiles the values. Although the experimental points of the four systems studied appeared very proximate at 293 K the position of the cloud points were significantly different between solvents (i.e., PVDF/DMAc/water vs. PVDF/NMP/water and PES/DMAc/water vs. PES/NMP/water) and between polymers (i.e., PVDF/DMAc/water vs. PES/DMAc/water and PVDF/NMP/water vs. PES/NMP/water). Cloud point experimental data obtained between 293 and 295 K (room temperature) are widely reported in the literature for the four systems studied [7,14,28,33,34,35,36,37,38,39,40,41,42,43,44,45,46]. Experimental results of this study fall within the range of results previously reported at room temperature. However, it is remarkable the huge dispersion of data reported for PVDF systems, especially in the PVDF/NMP/water system [41,44]. A relationship between the different polymer molecular weight characteristics of the polymers used in these studies with the variation of the cloud points reported was not found. On the other hand, this could be more rationally attributed to the difficulty observed to determine the experimental change in turbidity for PVDF systems compared to the clear change in PES systems, for which literature cloud points are more consistent [35,36].

For PVDF and PES systems, a significant influence of the temperature that displaces the cloud points to the right is observed. However, for PES systems, the displacement of the cloud point curves with the temperature was smaller than for PVDF. PES is classified as a glassy polymer with glass transition temperature (*T_g_* = 489–505 K) above the typical temperature used in phase inversion processes (298–333 K), while PVDF is in rubbery solid state (*T_g_* = 206–278 K) at this range of processing temperatures. As the cloud points represent a pseudo stable liquid phase point in the proximities to solid precipitation, this difference can be attributed to the different state of the polymers in the solid phase, being PVDF a rubbery-polymer and PES a glassy-polymer. The specific volume of the glassy polymers suffers a thermal expansion when temperature rises but there is a characteristic rigidity of polymer chains. In the rubbery state, there is a higher impact of the temperature on the increase of the specific volume, as chain mobility is more important than for glassy polymers [2]. Hence, the penetration of the solvents and therefore the solubility might be more relevant for polymers in the rubbery state (PVDF) than for glassy polymers (PES). These experimental results indicate that, from the thermodynamic point of view, tuning the NIPS process temperature could be considered an interesting approach to tailor the membrane morphology of rubbery polymers (such as PVDF). However, it might not be a reasonable choice for membranes prepared of glassy polymers such as PES.

### 4.2. Mathematical Modeling

The obtained solubility parameters at different temperatures are compiled in Table A4, Appendix A. It can be seen for all the components that the solubility decreased with the temperature. This reduction of the solubility parameters will affect the values of *χ_ij_* used in the simulations, so it is confirmed the necessity of calculating them considering the effect of temperature.

**Figure 4:**
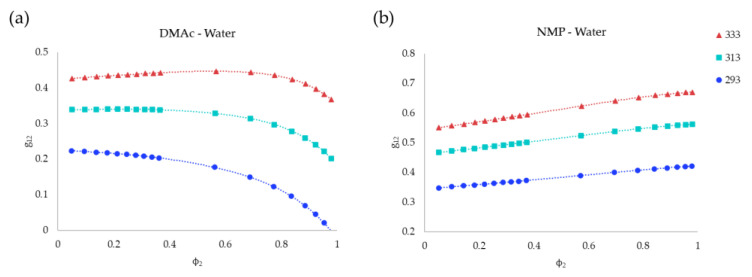
Influence of solvent concentration in *g_12_* binary interaction parameter at 293, 313 and 333 K for (**a**) DMAc–water and (**b**) NMP–water binary systems.

Figure 4 presents the *g_12_* curves as a function of solvent concentration (*ϕ_2_*) for the binary systems (a) DMAc–water and (b) NMP–water calculated using UNIFAC-Dortmund methodology. Table 5 presents the parameters that results from the fitting of the calculated *g_12_* values to the polynomial Equation (19). It can be seen that the interaction between DMAc and water decreased with the solvent concentration, while the NMP–water behaved as the opposite. Additionally, an increase of the *g_12_* values with the temperature is observed in both cases.

When the value of *g_12_* increases, the interaction between the solvent and non-solvent is lower, favoring the displacement of the binodal curve to the polymer–non-solvent axis, and therefore extending the region of homogeneous liquid phase [47]. In Figure 4, it can be seen that, overall, NMP-water *g_12_* presents higher values than for DMAc–water systems (worse interaction). Moreover, for the system DMAc–water, the interaction was favoured at increasing concentrations of DMAc and at lower temperatures, while for the NMP–water system, the high NMP concentrations and high temperatures decreased the interaction between the components. Accordingly, Figure 3 shows the expected displacement to the right of the cloud points with the temperature, and the larger liquid homogeneous region for the NMP systems.

#### 4.2.1. Modeling Binodal Curves not Considering the Contribution of the Ternary Interaction Term

**Figure 5:**
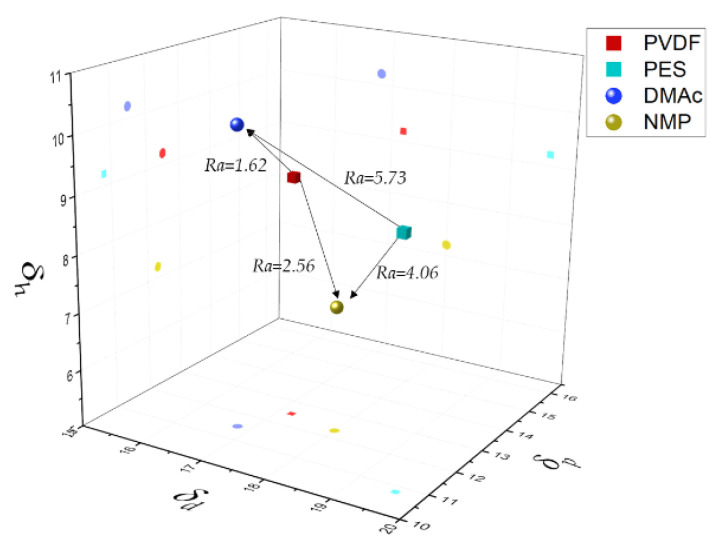
Hansen space and radius of interaction (*Ra*) according to ${\left(Ra\right)}^{2}\;=\;4{\left(\delta_{d,2}{-\delta }_{d,1}\right)}^{2}+{\left(\delta_{p,2}{-\delta }_{p,1}\right)}^{2}+{\left(\delta_{h,2}{-\delta }_{h,1}\right)}^{2}$ [23].

In this section it is considered a negligible effect of the ternary interaction parameter on the phase diagram (*χ_123_*= 0). This modeling approach has been the most widely reported in the literature [17,18,27,48]. Firstly, *χ_13_* and *χ_23_* parameters were calculated according to Equation (11) considering *α_13_ = α_23_* = 1 (Table 6). It can be seen that all *χ_13_* values decreased with temperature. On the other hand, *χ_23_* values increased with temperature for all binary systems except for PVDF/NMP. Figure 5 shows the solubility parameters in the Hansen space and the radius of interaction between each pair of compounds. Interestingly, PVDF was closer to the solvents than PES, especially PVDF was more soluble in DMAc (*Ra* = 1.62). Besides, PES was closer to NMP than to DMAc (*Ra* = 4.06). The solvation could be easier for PVDF since its monomer size was smaller compared with PES, and on the other hand the phenyl groups in PES polymer contribute to the steric hindrance.

**Figure A1:**
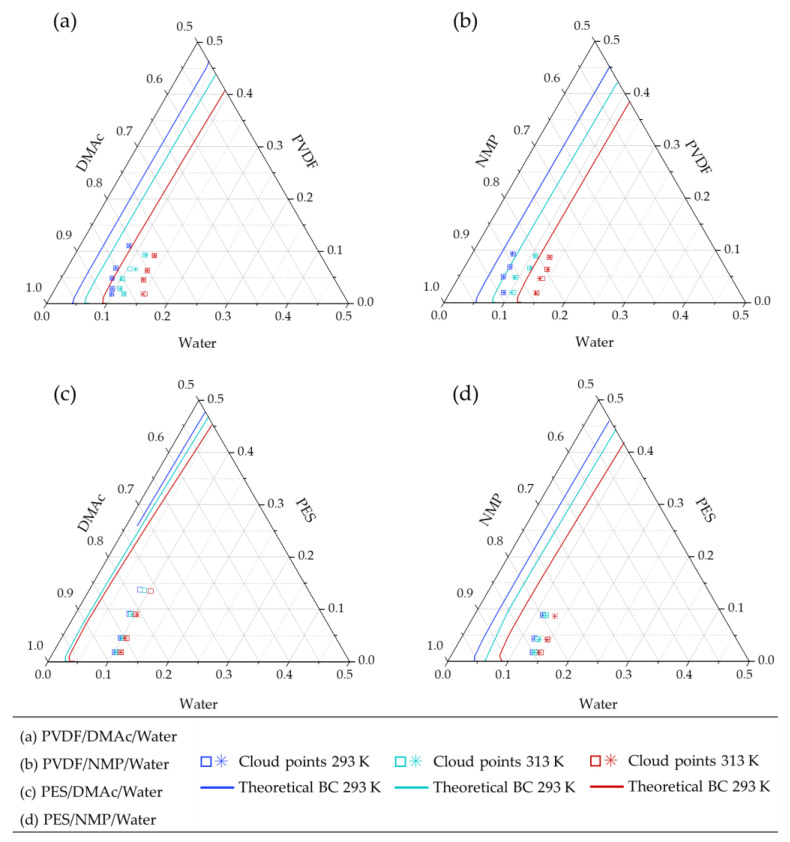
Cloud point and theoretical curves at 293, 313 and 333 K of (**a**) PVDF–DMAc–water, (**b**) PVDF–NMP–water, (**c**) PES–DMAc–water and (**d**) PES–NMP–water systems (α_13_ = α_13_ = 1 and *χ*_123_ = 0).

Figure A1 of Appendix A depicts the modeled binodal curves of ternary systems obtained under the above assumptions (i.e., neglecting the ternary interaction parameter). Overall, the model correctly predicts the displacement of the binodal curve to the right at increasing temperature, as it happens with the experimental data. However, the simulated curves did not fit adequately of experimental points. It is observed that all binodal curves were shifted to the left (solvent–polymer axis) in contrast to the experimental cloud points.

**Figure 6:**
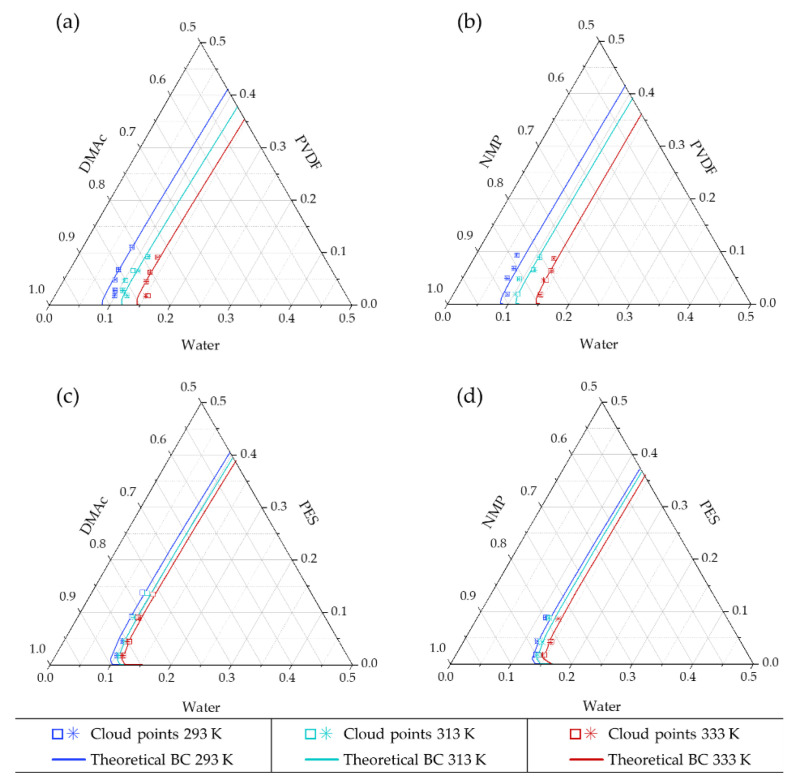
Cloud points and corrected theoretical binodal curves (BC) at 293, 313 and 333 K of (**a**) PVDF–DMAc–water, (**b**) PVDF–NMP–water, (**c**) PES–DMAc–water and (**d**) PES–NMP–water systems. Duplicated experimental results are represented by □ and *.

Different aspects, such as the polymer having a broad molecular weight distribution, or swelling or plasticizing effects occurring between the polymer and the solvent for binary mixtures have been reported to alter significantly the values of the Flory–Huggins binary interaction parameters [48,49,50]. Previous works have reported the use of a correction factor *α_ij_* in the calculation of binary interaction parameters *χ_13_* and *χ_23_*, Equation (11), to amend the deviation observed between the modeled and experimental cloud points. Therefore, a fitting procedure to estimate the correction factors was used obtaining the *χ_13_* and *χ_23_* values presented in Table 7. Simulated and experimental curves using this approach are depicted in Figure 6. Due to the fitting approach, the simulated curves are now fairly predicting the experimental points, as expected.

Table 7 shows that for PVDF systems the *α_13_* correction factors were near to 1, and *χ_23_* calculation did not require of a correction factor (*α_23_* = 1). For *χ_13_* a linear tendency with the temperature is observed with a slope of 0.0033 for both PVDF/DMAc and PVDF/NMP systems. Instead, PES systems needed significant correction in both *χ_13_* and *χ_23_* binary interaction parameters. The value of *α_13_* is the same for both PES systems and it increased with temperature. However, the solvent–polymer interaction, represented by *χ_23_*, needs a higher correction with *α_23_* values between 0.1 and 0.5. 

Other authors propose different *α_ij_* values. Lindving et al. [51] consider a general value of 0.6, independent of the system. Wei et al. [27] estimated fitting values of *α_13_* = 0.83 and *α_23_* = 0.08 for both PES/NMP/water and PES/DMAc/water systems at 298 K; these values differ from the results obtained in this study because of the revised expression of *g_12_* and the solubility parameters for PES employed in the present work. Although the model adequately predicts the experimental points, the use of correction factors entails important limitations and concerns: (1) low polymer concentration points are not well adjusted to the binodal curve; (2) two fitting parameters are needed (*α_13_* and *α_23_*) and (3) no reasonable relation is found in the adjustment of the alpha correction factors, thus limiting its extrapolation to other systems or finding a tendency. In this study, the necessity of a *α_23_* correction factor for PES systems may be attributed to a higher cosolvency effect between the three components, this phenomenon will be studied in detail in the following section. According to Barth et al. [52], who studied PES/DMF/water systems, the use of *α_13_* in the calculation of *χ_13_* points to the occurrence of ternary interactions between polymer, solvent and non-solvent. This approach, that is described below, has not been explored so far for the present ternary systems.

#### 4.2.2. Modeling Binodal Curves Considering the Contribution of the Ternary Interaction Term

**Figure 7:**
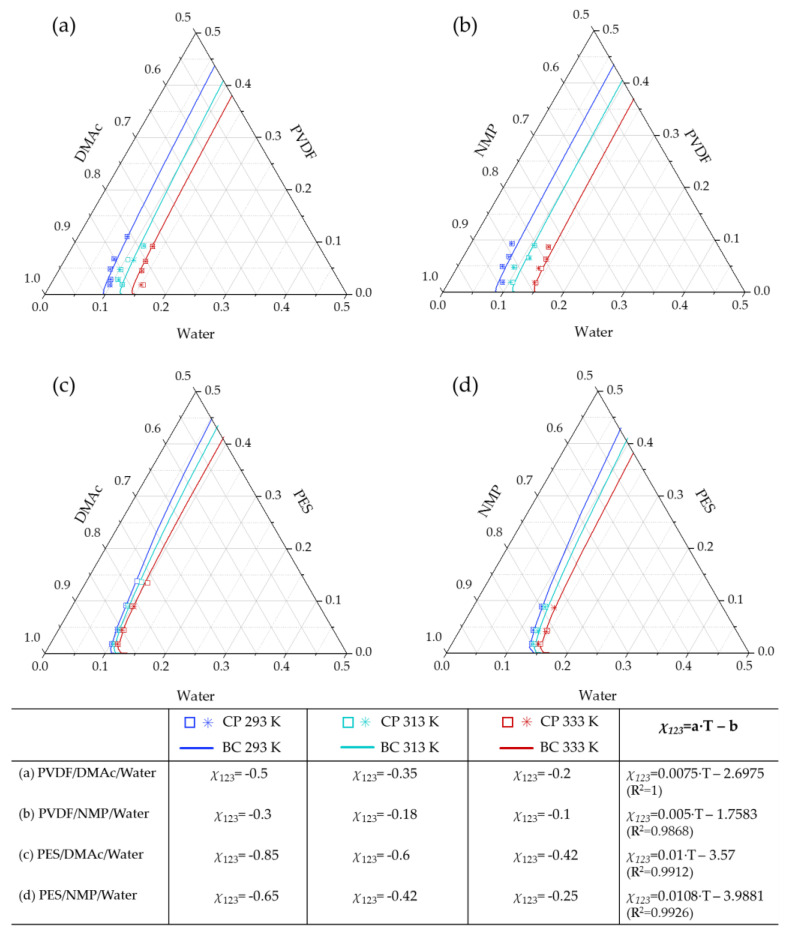
Binodal curves using adjusted *χ_123_* interaction parameter (**a**) PVDF–DMAc–water, (**b**) PVDF–NMP–water, (**c**) PES–DMAc–water and (**d**) PES–NMP–water systems. Duplicated experimental results are represented by □ and *.

To investigate the influence of the ternary interaction term of the systems, the *χ_123_* ternary interaction parameter was included in the thermodynamic model. Given the lack of any methodology reported in the literature to calculate the ternary interaction parameter (*χ_123_*), we decided to estimate it by adjusting the experimental cloud point data to the model, Equations (4)–(6). Figure 7 presents the values of *χ_123_* and the resulting simulated binodal curves. 

It can be seen that the fitted *χ_123_* values were negative indicating a strong interaction between the components. This ternary interaction was not considered in previous literature approaches to the systems studied, where only binary interactions were deemed. Few authors have taken into account this parameter for polycaprolactone/dimethylformamide/water system [15] and poly(ethylene-co-vinyl alcohol) (EVAL)/2-propanol (2P)/water system [53]. The negative sign of *χ_123_* implies a cosolvency between the solvent and water. Cosolvency is a phenomenon that has been broadly reported in ternary systems where polymers were soluble on binary mixtures of two non-solvents EVAL/2P/water and polystyrene/acetone/diethylether [53,54]. In the present study, it is hypothesized that at certain range of solvent/non-solvent compositions, the solubility of the polymer is higher than expected simply considering binary interaction between the components, which can be attributed to a cosolvency effect (solvent/non-solvent mixtures acting as a solvent). Higher absolute values of *χ_123_* means higher cosolvency influence. PES presents higher degree of cosolvency (higher *χ_123_* estimated values) than PVDF systems, probably due to its worse solubility on the solvents (Figure 5).

It is observed a linear trend of *χ_123_* with temperature, in the range 293–333 K, as shown in the fitting equations inserted in Figure 6. The absolute χ*_123_* value follows the order PVDF/NMP/water > PVDF/DMAc/water > PES/NMP/water > PES/DMAc/water. The increase of χ*_123_* with temperature indicates that cosolvency remains, although this effect is diminished with temperature as indicated by the reduction in the absolute values of χ*_123_*. Now the curves adjust better to the experimental cloud points even at low polymer concentrations.

The results obtained in the present study highlight that, despite being the most widely spread methodology, neglecting the ternary interaction among the components of the phase inversion process might be erroneous, particularly for polymers presenting low solubility in traditional solvents. It is also envisaged that the ternary interaction term would have an important contribution in describing the equilibrium of systems that incorporate solvents with low solubility properties, i.e., typical green solvents such as dimethylsulfoxide (DMSO) [55], PolarClean^®^ [56] or 2Pyr [6].

## 5. Conclusions

The influence of temperature in the processing of polymeric membranes by NIPS has not been sufficiently studied. In this work, four systems composed by PVDF and PES as the polymers, DMAc and NMP as the solvents and water as the non-solvent were studied at three temperatures (293, 313 and 333 K). The experimental cloud points of the systems were obtained and used to validate a mathematical model based on the Flory–Huggins theory that included the effect of the temperature.

The experimental cloud points showed that as the temperature increased the solubility region of the ternary systems was enlarged. The displacement of the binodal curve toward the polymer/non-solvent axis was more evident for the PVDF (rubbery polymer) than in the PES (glassy polymer) systems.

For the first time, the thermodynamic model developed in the present work successfully incorporated the effect of temperature to predict the binodal curves of four polymer/solvent/water ternary systems. We found that the model considering only binary interaction parameters critically deviated from experimental data. Therefore, we evaluated the incorporation to the model of a ternary interaction term that includes a ternary interaction parameter *χ*_123_, which was estimated for each system by fitting the experimental data to the proposed model. Negative values of *χ_123_* indicated the strong interaction between the three components and the presence of a cosolvency phenomenon that increased the homogeneous region where the polymer is still soluble. In all systems, the magnitude of the ternary interaction parameter decreased linearly at increasing temperatures. 

The main disadvantage of the implementation of the ternary interaction term in the model is that *χ_123_* parameter must be estimated by fitting to experimental data. To expand the universal application of this model, future works should aim at broadening the experimental validation with other polymer systems and the search of methodologies to estimate reliable *χ_123_* values for different ternary systems.

## Figures and Tables

**Table 1:** Chemical structure and relevant properties of solvents and non-solvents. Data at 298 K are reference (ref) values.

Solvent	Formula	Structure	*M_w_* (g/mol)	*T* (K)	*ρ* (g/cm^3^)	*V_i_* (cm^3^/mol)
DMAc	C_4_H_9_NO	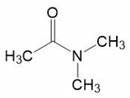	87.12	298	0.9365	93.031
				293	0.9408	92.599
				313	0.9233	94.355
				333	0.9054	96.224
NMP	C_5_H_9_NO	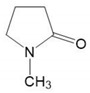	99.13	298	1.0262	96.600
				293	1.0302	96.231
				313	1.0141	97.754
				333	0.9977	99.357
Water	H_2_O	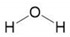	18.01	298	0.9945	18.114
				293	0.9965	18.079
				313	0.9888	18.219
				333	0.9797	18.388

**Table 2:** Hansen’s solubility parameters at 298 K [23].

	*δ_d_* (MPa)^1/2^	*δ_p_* (MPa)^1/2^	*δ_h_* (MPa)^1/2^	*δ_T_* (MPa)^1/2^
PVDF	17.2	12.5	9.2	23.167
PES	19.6	10.8	9.2	24.196
DMAc	16.8	11.5	10.2	22.771
NMP	18	12.3	7.2	22.959
Water	15.5	16	42.3	47.807

**Table 3:** Chemical structure and properties of monomer units of the polymers.

Polymer	Formula	Structure	*ρ* (g/cm^3^)	M_w_* (g/mol)	*V_w_* (cm^3^/mol)	*T* (K)	V_i_* (cm^3^/mol)
PVDF	C_2_H_2_F_2_	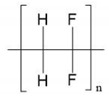	1.76	64.03	25.03	298	35.896
						293	35.839
						313	36.064
						333	36.290
PES	C_12_H_8_SO_3_	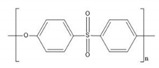	1.38	232.25	112.15	298	190.997
						293	190.745
						313	191.754
						333	192.763

* Molecular weight and molar volume of the monomer unit.

**Table 4:** Calculated physical parameters and fitting parameters use in the calculation procedure.

Calculated Parameters		Fitted Parameters	
*χ_13_*	Equation (9)	*α_13_*	Equation (9)
*χ_23_*	Equation (9)	*α_23_*	Equation (9)
*g_12_*	Equation (19)	*χ_123_*	Equations (4)–(6)

**Table 5:** Coefficient of solvent/non-solvent interaction parameters for the equation $g_{12}\left(\phi_{2}\right){\;=\;a}_{o}{+a}_{1}\phi_{2}{+a}_{2}\phi_{2}^{2}{+a}_{3}\phi_{2}^{3}{+a}_{4}\phi_{2}^{4}{+a}_{5}\phi_{2}^{5}{+a}_{6}\phi_{2}^{6}$.

System	T (K)	*a*_0_	*a*_1_	*a*_2_	*a*_3_	*a*_4_	*a*_5_	*a*_6_
DMAc/Water	293	0.2265	−0.0758	0.2781	−1.1099	1.4731	−0.815	
	313	0.3396	−0.0269	0.3184	−1.1455	1.5387	−0.8392	
	333	0.4223	0.0945	−0.358	1.5048	−3.2626	3.316	−1.3648
NMP/Water	293	0.3442	0.0751	−0.0107	0.0549	−0.0411		
	313	0.4617	0.1069	−0.0234	0.0871	−0.0693		
	333	0.5438	0.1373	−0.0365	0.1273	−0.1003		

**Table 6:** Binary interaction parameters (*χ_13_* and *χ_23_*) at different temperatures from solubility parameters with *α_13_ = α_13_ =* 1 and *χ_123_*= 0.

Temperature (K)	293		313		333	
Interaction Parameter	*χ* *_13_*	*χ* *_23_*	*χ* *_13_*	*χ* *_23_*	*χ* *_13_*	*χ* *_23_*
PVDF/DMAc/Water	2.106	0.023	1.879	0.031	1.684	0.043
PVDF/NMP/Water	2.106	0.069	1.879	0.053	1.684	0.042
PES/DMAc/Water	2.237	0.299	2.003	0.337	1.802	0.380
PES/NMP/Water	2.237	0.157	2.003	0.174	1.802	0.195

**Table 7:** Corrected binary interaction parameters *χ_ij_* with the corresponding correction constants *α_ij_.*

	293 K				313 K				333 K			
	*α_13_*	*χ* *_13_*	*α_23_*	*χ* *_23_*	*α_13_*	*χ* *_13_*	*α_23_*	*χ* *_23_*	*α_13_*	*χ* *_13_*	*α_23_*	*χ* *_23_*
PVDF/DMAc/Water	0.7	1.474	1	0.0234	0.75	1.409	1	0.0307	0.83	1.397	1	0.0429
PVDF/NMP/Water	0.8	1.685	1	0.069	0.87	1.635	1	0.053	0.93	1.566	1	0.042
PES/DMAc/Water	0.64	1.432	0.1	0.030	0.75	1.502	0.1	0.034	0.85	1.531	0.2	0.076
PES/NMP/Water	0.64	1.432	0.5	0.078	0.75	1.502	0.5	0.087	0.85	1.531	0.5	0.098

## Data Availability

The data presented in this study are available on request from the corresponding author.

## Appendix A

The UNIFAC modified (Dortmund) methodology is a way to obtain activity coefficient of binary mixtures from group contribution data.

Firstly, contribution groups are assigned to the compounds involved in the mixture. For a multitude of compounds this step can be easily done with the Dortmund data bank tool of assignation http://www.ddbst.com/unifacga.html (accessed on 7 September 2020). Secondly, volume and surface area data of each group and the binary interaction between all the groups involved in a mixture are compiled from http://www.ddbst.com/PublishedParametersUNIFACDO.html (accessed on 7 September 2020).

Then the modified Dortmund UNIFAC methodology is applied. The activity coefficient γ of each compound is calculated using Equation (A1), this equation is composed by the combinatorial part Equation (A2) and the residual part Equation (A3).

	$\ln\gamma_{i}=\ln\gamma_{i}^{c}+\ln\gamma_{i}^{R}$	(A1)
Combinatorial	$\ln\gamma_{i}^{c}=1-V_{i}^{\prime }+\ln V_{i}^{\prime }-5\;q_{i}\left(1-\frac{V_{i}}{F_{i}}+\ln\frac{V_{i}}{F_{i}}\right)$	(A2)
Residual $\Gamma_{k}^{\left(i\right)}$: residual activity coefficient of group k in a reference solution containing molecules of type i	$\ln\gamma_{i}^{R}=\underset{k}{\sum }v_{k}^{\left(i\right)}\left[\ln\Gamma_{K}-\ln\Gamma_{K}^{\left(i\right)}\right]$	(A3)
$\Gamma_{k}$: group residual activity coefficient	$\ln\Gamma_{k}=Q_{k}\left[1-\ln\left(\sum_{m}\left(\Theta_{m}\Psi_{mk}\right)\right)-\sum_{m}\frac{\Theta_{m}\Psi_{km}}{\sum_{n}\Theta_{n}\Psi_{nm}}\right]$	(A4)
Surface area/mole fraction ratio	$F_{i}=\frac{q_{i}}{\sum_{j}q_{j}x_{j}}$	(A5)
Volume/mole fraction ratio	$V_{i}=\frac{r_{i}}{\sum_{j}r_{j}x_{j}}$	(A6)
	$V_{i}^{\prime }=\frac{r_{i}^{3/4}}{\sum_{j}r_{j}^{3/4}x_{j}}$	(A7)
Volume of the pure component (i)	$r_{i}=\underset{k}{\sum }v_{k}^{\left(i\right)}R_{k}$	(A8)
Surface area of the pure component (i)	$q_{i}=\underset{k}{\sum }v_{k}^{\left(i\right)}Q_{k}$	(A9)
Area fraction of group (m)	$\Theta_{m}=\frac{Q_{m}X_{m}}{\sum_{n}Q_{n}X_{n}}\;$	(A10)
Mole fraction of group m in the mixture	$X_{m}=\frac{\sum_{j}v_{m}^{\left(i\right)}x_{j}}{\sum_{j}\sum_{n}v_{n}^{\left(i\right)}x_{j}}\;$	(A11)
Group interaction parameter	$\;\Psi_{mn}=exp\left(-\frac{a_{mn}+b_{mn}T+c_{mn}T^{2}}{T}\right)$	(A12)

Table A1 compiles the information of the group assignment and volume (*R_k_*) and area numbers (*Q_k_*) for the compounds used in this study. Table A2 presents the interaction parameters between the groups listed in Table A1 [31,57].

**Table A1:** Group assignment, volume and area numbers of different compounds based on UNIFAC-Dortmund methodology.

Compound	Formula	Structure	GROUP Assignment		Q_k_	R_k_
			Group × V_ik_	Group-Sub Group No.		
DMAc	C_4_H_9_NO		CH_3_ × 1	1-1	1.0608	0.6325
			CON(CH_3_)_2_ × 1	48-101	1.9643	2.4748
NMP	C_5_H_9_NO		cy-CH_2_ × 3	42-78	0.8635	0.7136
			cy-CON-CH_3_ × 1	46-86	3.2	3.981
Water	H_2_O		H_2_O × 1	7	2.4561	1.7334

**Table A2:** Interaction parameters between UNIFAC groups.

	n,m	a_nm,1_	a_nm,2_	a_nm,3_	a_mn,1_	a_mn,2_	a_mn,3_
Water–DMAc	1,7	1391.3	−3.6156	0.001144	−17.253	0.8389	0.000902
	1,48	1529.52	−6.2025	0.00975	82.6	−0.615	−0.00062
	7,48	64.43999	−0.0094	-	−439.58	0.3142	-
Water–NMP	7,42	274.37	−0.5861	−0.0003	1632.9	−2.8719	0.003455
	7,46	659.22	−1.8841	-	−588.21	0.9707	-
	42,46	298.46	−0.6823	-	499.59	−0.8158	-

**Table A3:** Average values of cloud point data at the three temperatures for the four studied systems.

	**Φ_PVDF_**			**Φ_DMAc_**			**Φ_Water_**		
293 K	0.0183	±	2.00 × 10^5^	0.8837	±	5.90 × 10^4^	0.0988	±	1.95 × 10^4^
	0.0286	±	1.00 × 10^5^	0.8769	±	2.15 × 10^4^	0.0946	±	2.25 × 10^4^
	0.0482	±	5.00 × 10^6^	0.8673	±	6.00 × 10^5^	0.0845	±	5.50 × 10^5^
	0.0677	±	2.00 × 10^5^	0.8516	±	8.50 × 10^5^	0.0808	±	1.05 × 10^4^
	0.1103	±	9.00 × 10^5^	0.8084	±	8.50 × 10^5^	0.0813	±	1.75 × 10^4^
313 K	0.0184	±	1.05 × 10^5^	0.8626	±	1.25 × 10^4^	0.1190	±	2.30 × 10^4^
	0.0282	±	4.50 × 10^5^	0.8645	±	4.30 × 10^4^	0.1073	±	4.80 × 10^4^
	0.0473	±	6.50× 10^5^	0.8512	±	1.17 × 10^3^	0.1016	±	1.24 × 10^3^
	0.0656	±	3.45 × 10^4^	0.8247	±	4.55 × 10^3^	0.1098	±	4.89 × 10^3^
	0.0926	±	2.60 × 10^4^	0.7904	±	6.80 × 10^4^	0.1169	±	4.20 × 10^4^
333 K	0.0180	±	4.00 × 10^5^	0.8297	±	1.93× 10^3^	0.1523	±	1.97 × 10^3^
	0.0449	±	0.00 × 10^0^	0.8172	±	0.00 × 10^0^	0.1379	±	0.00 × 10^0^
	0.0630	±	0.00 × 10^0^	0.8017	±	0.00 × 10^0^	0.1354	±	0.00 × 10^0^
	0.0915	±	0.00 × 10^0^	0.7756	±	0.00 × 10^0^	0.1329	±	0.00 × 10^0^
	**Φ_PVDF_**			**Φ_NMP_**			**Φ_Water_**		
293 K	0.0192	±	1.00 × 10^5^	0.8917	±	2.45 × 10^4^	0.0892	±	2.55 × 10^4^
	0.0491	±	5.00 × 10^6^	0.8771	±	1.25 × 10^4^	0.0738	±	1.35 × 10^4^
	0.0680	±	3.00 × 10^5^	0.8565	±	1.80 × 10^4^	0.0755	±	2.05 × 10^4^
	0.0929	±	2.05 × 10^4^	0.8392	±	2.90 × 10^4^	0.0679	±	4.95 × 10^4^
313 K	0.0189	±	3.00 × 10^5^	0.8767	±	1.73 × 10^3^	0.1044	±	1.77 × 10^3^
	0.0480	±	4.00 × 10^5^	0.8576	±	7.30 × 10^4^	0.0944	±	7.75 × 10^4^
	0.0655	±	7.00 × 10^5^	0.8245	±	7.30 × 10^4^	0.1101	±	8.05 × 10^4^
	0.0890	±	2.10 × 10^4^	0.8039	±	4.15 × 10^4^	0.1071	±	6.20 × 10^4^
333 K	0.0180	±	2.00 × 10^5^	0.8378	±	1.07 × 10^3^	0.1442	±	1.09 × 10^3^
	0.0457	±	1.40 × 10^4^	0.8161	±	2.44 × 10^3^	0.1382	±	2.58 × 10^3^
	0.0633	±	8.00 × 10^5^	0.7974	±	8.55 × 10^4^	0.1393	±	9.30 × 10^4^
	0.0865	±	1.65 × 10^4^	0.7816	±	4.50 × 10^5^	0.1318	±	2.10 × 10^4^
	**Φ_PES_**			**Φ_DMAc_**			**Φ_Water_**		
293 K	0.0179	±	2.00 × 10^5^	0.8798	±	6.50 × 10^5^	0.1023	±	9.00 × 10^5^
	0.0450	±	7.00 × 10^5^	0.8566	±	4.90 × 10^4^	0.0984	±	5.60 × 10^4^
	0.0912	±	3.65 × 10^4^	0.8188	±	7.50 × 10^4^	0.0900	±	1.11 × 10^3^
	0.1375	±	0.00 × 10^0^	0.7787	±	0.00 × 10^0^	0.0838	±	0.00 × 10^0^
313 K	0.0178	±	3.50 × 10^5^	0.8759	±	6.45 × 10^4^	0.1063	±	6.80 × 10^4^
	0.0448	±	8.00 × 10^5^	0.8533	±	7.85 × 10^4^	0.1018	±	8.70 × 10^4^
	0.0908	±	3.80 × 10^4^	0.8156	±	9.20 × 10^4^	0.0936	±	1.30 × 10^3^
	0.0684	±	0.00 × 10^0^	0.7751	±	0.00 × 10^0^	0.0881	±	0.00 × 10^0^
333 K	0.0177	±	2.50 × 10^5^	0.8703	±	3.05 × 10^4^	0.1120	±	3.30 × 10^4^
	0.0446	±	1.05 × 10^4^	0.8483	±	1.14 × 10^3^	0.1071	±	1.24 × 10^3^
	0.0901	±	3.55 × 10^4^	0.8086	±	7.50 × 10	0.1013	±	1.11 × 10^3^
	**Φ_PES_**			**Φ_NMP_**			**Φ_Water_**		
293 K	0.0174	±	4.00 × 10^5^	0.8511	±	9.20 × 10^4^	0.1315	±	9.60 × 10^4^
	0.0429	±	7.55 × 10^4^	0.8341	±	3.00 × 10^4^	0.1230	±	1.06 × 10^3^
	0.0890	±	5.25 × 10^4^	0.8025	±	3.68 × 10^3^	0.1135	±	8.25 × 10^4^
313 K	0.0173	±	4.00 × 10^5^	0.8468	±	8.75 × 10^4^	0.1359	±	9.10 × 10^4^
	0.0426	±	7.75 × 10^4^	0.8286	±	6.90 × 10^4^	0.1287	±	1.46 × 10^3^
	0.0881	±	0.00 × 10^0^	0.7926	±	0.00 × 10^0^	0.1194	±	0.00 × 10^0^
333 K	0.0171	±	5.00 × 10^5^	0.8382	±	1.46 × 10^3^	0.1447	±	1.50 × 10^3^
	0.0419	±	6.70 × 10^4^	0.8138	±	1.05 × 10^3^	0.1444	±	3.80 × 10^4^
	0.0863	±	0.00 × 10^0^	0.7792	±	0.00 × 10^0^	0.1345	±	0.00 × 10^0^

**Table A4:** Solubility parameters calculated at different temperatures from Hansen solubility parameters at 298 K.

T (K)	δ_d_ (MPa^1/2^)	δ_p_ (MPa^1/2^)	δ_h_ (MPa^1/2^)	δ_T_ (MPa^1/2^)
Polymer–PVDF				
298	17.2	12.5	9.2	23.167
293	17.234	12.510	9.264	23.223
313	17.099	12.471	9.012	23.003
333	16.967	12.432	8.767	22.788
Polymer–PES				
298	19.6	10.8	9.2	24.196
293	19.632	10.807	9.262	24.249
313	19.503	10.779	9.015	24.038
333	19.376	10.750	8.775	23.833
Solvent–DMAc				
298	16.8	11.5	10.2	22.771
293	16.898	11.527	10.286	22.896
313	16.506	11.419	9.945	22.399
333	16.106	11.308	9.610	21.900
Solvent–NMP				
298	18	12.3	7.2	22.959
293	18.086	12.324	7.258	23.058
313	17.735	12.227	7.028	22.659
333	17.378	12.128	6.803	22.257
Non-solvent–Water				
298	15.5	16	42.3	47.807
293	15.538	16.015	42.600	48.090
313	15.388	15.954	41.413	46.972
333	15.211	15.880	40.228	45.846
